# Factors associated with the occurrence of MRSA CC398 in herds of fattening pigs in Germany

**DOI:** 10.1186/1746-6148-7-69

**Published:** 2011-11-10

**Authors:** Katja Alt, Alexandra Fetsch, Andreas Schroeter, Beatriz Guerra, Jens A Hammerl, Stefan Hertwig, Natalja Senkov, Anna Geinets, Christine Mueller-Graf, Juliane Braeunig, Annemarie Kaesbohrer, Bernd Appel, Andreas Hensel, Bernd-Alois Tenhagen

**Affiliations:** 1 Federal Institute for Risk Assessment, Thielallee 88-92, D-12277 Berlin, Germany

## Abstract

**Background:**

The purpose of this study was to investigate the prevalence of MRSA in herds of fattening pigs in different regions of Germany, and to determine factors associated with the occurrence of this pathogen. For this purpose pooled dust samples were collected, and a questionnaire covered information regarding herd characteristics and management practices. Samples were pre-enriched in high-salt medium followed by selective enrichment containing cefoxitin/aztreonam, and culturing. Presumptive colonies were confirmed by multiplex-PCR targeting *nuc-*, *mecA*- and 16S rRNA-genes. Isolates were s*pa- *and SCC*mec*-, and in selected cases, multilocus sequence-typed. Susceptibilities to 13 antimicrobials were determined by broth microdilution. Statistical analysis was carried out using backward stepwise logistic regression to calculate odds ratios with the MRSA test result as the outcome and herd characteristics as categorical covariates.

**Results:**

Overall, 152 of 290 (52%) fattening pig farms tested positive for MRSA. The prevalence in the east, north- and south-west of Germany ranged from 39 to 59%.

t011 (66%) and t034 (23%) were the most commonly identified s*pa*-types, and 85% of isolates carried SCC*mec *Type V. Identified *spa*-types were all associated with clonal complex CC398. Susceptibility testing revealed that all isolates were resistant to tetracycline. High resistance rates were also found for sulfamethoxazole/trimethoprim (40%), and quinupristin/dalfopristin (32%). In addition, 83% of strains displayed multidrug resistant (> 3 substance classes) phenotypes.

Logistic regression revealed herd size (large farms OR: 5.4; CI: 2.7-11.2; p < 0.05), and production type (wean-to-finish OR: 4.0; CI: 1.6-10.4; p < 0.05) as risk factors associated with a positive MRSA finding in fattening pig operations.

**Conclusions:**

MRSA CC398 is widely distributed among herds of fattening pigs in Germany. Farm management plays a crucial role in the dissemination of MRSA with herd size, and production type representing potential major indicators.

## Background

In recent years, the emergence of the MRSA multilocus sequence type (MLST) CC398 has been reported in livestock in Europe and North America mainly in pigs, but also in veal calves and poultry [1-4]. Characteristic for this newly identified type are its wide spread among livestock and persons in close contact with colonised animals, and its low morbidity.

Animal disease involving MRSA CC398 has been described, especially in horses [5-7] and dairy cattle [8]. In addition, CC398 has been associated with severe diseases in humans [9-12]. Currently the only route of transmission considered playing a relevant role in the transmission of MRSA CC398 from animals to humans is direct contact with colonised livestock [13]. Cross-transmission by contact between humans and pigs or veal calves led to the classification of persons occupationally exposed to pigs or veal calves, e. g. farmers, veterinarians and slaughterhouse staff as high risk population for the carriage of MRSA CC398 [14,15].

Typically, livestock isolates display a variety of multiresistant profiles, including resistance to β-lactams and tetracycline in most of them. It has been shown, that phenotypic resistance to several antimicrobials largely used in clinical practice is encoded by genes located on mobile elements, which reflects the potential for spread and acquisition of new traits, and the relevance of monitoring [16,17].

In order to evaluate the risk posed to humans, and to assess possible routes of transmission, several studies have been undertaken to estimate the prevalence of MRSA CC398 focussing on the pig population as a starting point. At herd level, prevalence of MRSA CC398 has been reported with 45% positive farms in North America, and ranging between 0 and 46% among breeding holdings in European Union Member States [2,4,18]. Data from The Netherlands showed 39% prevalence at pig and 81% at batch level, when pigs at slaughter were tested [1]. In Germany, the Federal Institute for Risk Assessment (BfR) conducted in 2007 in cooperation with two federal states a survey in abattoirs revealing that up to 71% of 520 pigs, and 51 of 52 batches were MRSA positive [19]. In 2008, 201 breeding pig herds distributed across Germany were examined in the framework of an EU-wide survey according to Commission Decision 2008/55/EC. Of those, 42% tested positive [20]. The purpose of the present study was to estimate of the prevalence of MRSA CC398 in dust samples taken in the finishing compartments in German fattening pig farms of different types. Additionally, we assumed that regional differences in the management structures should be reflected in a different MRSA prevalence among fattening herds. To assess these hypotheses we examined fattening pig farms of different management structures located in distinct regions of Germany and determined potential risk factors for the dissemination of MRSA CC398. The current study provides additional information regarding the distribution of MRSA CC398 among swine population, and describes molecular and phenotypic resistance characteristics of collected isolates. Furthermore, factors that potentially play key roles in the spread of this pathogen among swine are outlined.

## Results

### Prevalence of MRSA

A total of 290 operations housing more than 100 finishers distributed across seven federal states agreed to take part in the survey. These operations were allocated to three regions. The north-western (NW) region was represented by 72, the eastern (E) by 65, and the south-west (SW) by 153 operations. A brief overview of the prevalence according to the main farm characteristics is provided in Table 1.

**Table 1:** Results of logistic regression for univariate and multivariate models Only cases with full information on the analysed variable or set of variables were included.

Variable	Category	**No**.	Positive %	Univariate			Multivariate		
				
				OR^a^	-95 % CI	+95% CI	OR^a^	-95 % CI	+95% CI
Region	East	65	39	Ref					
	Northwest	72	51	1.7	0.9	3.3	2.6	1.2	5.8
	Southwest	153	59	2.3	1.3	4.1	3.8	1.9	7.9
Herd size	Small	83	30	Ref					
	Medium	87	60	3.5	2.7	7.2	3.8	1.9	7.5
	Large	116	63	3.4	1.8	6.5	5.4	2.7	11
Production type	Farrow-to-finish	65	31	Ref					
	Wean-to-finish	38	63	3.9	1.7	9.0	4.0	1.6	10
	Grow-to-finish	185	58	3.1	1.7	5.6	2.0	1.0	4.0
Purchase of pigs	No purchase	73	36	Ref					
	Purchase from 1-2 sources	165	55	2.2	1.3	3.9			
	Purchase from multiple sources	49	71	4.5	2.1	9.9			
Animal flow system	Continuous	90	40	Ref					
	AI/AO^b ^with cleanup	22	46	1.3	0.5	3.2			
	AI/AO^b^with cleanup and disinfection	154	59	2.2	1.3	3.4			
Flooring	Concrete with bedding	25	16	Ref					
	Partially slatted	41	44	7.1	2.4	21			
	Totally slatted	219	58	4.1	1.2	14			
Cattle in the farm	Present	235	56	Ref					
	Absent	55	36	0.5	0.2	0.8			
Use of antimicrobials^c^	No	126	42	Ref					
	Yes	164	60	2.1	1.3	3.4			

Categories with the lowest MRSA prevalence were chosen as references (indicated as Ref).

^a ^p-value < 0.05

^b ^All in/all out

^c ^Reported treatments at group level

Overall, 152 (52%) herds tested positive for MRSA. The prevalence in the SW was 59%, 51% in the NW, and 39% in the E. The prevalence of MRSA varied at state level between 29 and 70% (data not shown).

### Molecular typing results

All isolates were assigned to the clonal complex CC398 by *spa*-typing. Altogether, twelve different *spa*-types were identified (Table 2). The vast majority of isolates belonged to types t011 (66%) and t034 (23%). One new *spa*-type, t5488, was detected. Isolates carried primarily (85%) SCC*mec-*type V followed by 11% type V* (a variant first identified as III) [21]. In one case type IVa was found, and five isolates were not typable after the method previously described [22]. The most frequent combinations of *spa*- and SCC*mec*-types were t011/V (63%), t034/V (13%) and t034/V* (9%) (Table 2).

**Table 2:** Distribution of spa-types and their combinations with identified SCC*mec*-types among 152 MRSA isolates

*Spa*-type	Number of isolates (%)	SCC*mec*-type V* (%)	SCC*mec*-type IVa (%)	SCC*mec*-type V (%)	SCC*mec *non typeable (%)
t011	101 (66)	1 (0.7)	1 (0.7)	96 (63)	3 (2.0)
t034	35 (23)	14 (9.2)		19 (13)	2 (1.4)
t108	4 (2.6)			4 (2.6)	
t571	2 (1.3)			2 (1.4)	
t1255	1 (0.7)			1 (0.7)	
t1451	3 (2.0)			3 (2.1)	
t1928	1 (0.7)	1 (0.7)			
t2383	1 (0.7)			1 (0.7)	
t2510	1 (0.7)			1 (0.7)	
t2582	1 (0.7)	1 (0.7)			
t2997	1 (0.7)			1 (0.7)	
t5488	1 (0.7)			1 (0.7)	
Total	152 (100)	17 (11)	1 (0.7)	129 (85)	5 (3.3)

MLST was performed once for *spa*-types t2997, t2582, t1451, t5488, and t2383 revealing in all cases sequence type ST398.

### Antimicrobial resistance profiles, and application of antimicrobials at group level

Susceptibility testing results are shown in Table 3. Overall, 45 resistance profiles were detected. Twenty two (15%) isolates displayed the most common resistance profile TET-OXA. Multidrug resistance (MDR) to a range of three to eight substance classes was found in 126 (83%) of strains. Among those, the most predominant MDR profiles were OXA-TET-ERY-CLI-QUI/DAL (13%), and OXA-TET-ERY-CLI-KAN-GEN-SXT-QUI/DAL (10%). Interestingly, the latter was exclusively associated with *spa-*type t011. Resistance to SXT was observed in 17 resistance profiles of which 16 were MDR profiles. QUI/DAL resistance occurred in 13 resistance profiles always associated with resistance to four or more substance classes (data not shown).

**Table 3:** Distribution of MIC values for MRSA CC398 identified in this study

	No. of isolates with MIC (mg/L)											Resistant (non-wild-type)^a^	
**Antimicrobial agent(s)**	**0.25**	**0.5**	**1**	**2**	**4**	**8**	**16**	**32**	**64**	**128**	**256**	**no**.	**%**

OXA + 2% NaCl	-	4	2	1	10	57	**78**	-	-	-	-	145	95
ERY	4	45	10	0	0	0	0	**93**	-	-	-	93	61
CLI	48	6	1	0	1	1	4	90	**1**	-	-	104	68
TET	-	-	0	0	0	0	0	1	37	**114**	-	152	100
CHL	-	-	-	0	2	58	82	4	4	2	0	10	6.6
GEN	-	57	48	14	4	3	3	6	4	**13**	-	33	22
KAN	-	-	-	-	-	77	23	15	10	3	**24**	75	49
CIP	-	134	10	2	3	3	0	0	0	-	-	8	5.3
SXT^b^	66	24	40	18	3	1	0	-	-	-	-	61	40
QUI/DAL	-	52	52	25	12	5	**6**	-	-	-	-	48	32
MUP	-	-	152	0	0	0	0	-	-	-	-	0	0.0
LZD	-	-	5	56	91	0	0	-	-	-	-	0	0.0
VAN	-	-	-	152	0	0	0	0	-	-	-	0	0.0

OXA: oxacillin, ERY: erythromycin, CLI: clindamycin, TET: tetracycline, CHL: chloramphenicol, GEN: gentamicin, KAN: kanamycin, CIP: ciprofloxacin, SXT: sulfamethoxazole/trimethoprim, QUI/DAL: quinupristin/dalfopristin, MUP: mupirocin, LZD: linezolid, and VAN: vancomycin.

^a ^Isolates were classified as resistant after EUCAST epidemiological cut-off values (black vertical lines) for MRSA and/or *S. aureus *valid at the time of submission.

^b ^The MIC values of sulfamethoxazole/trimethoprim (19:1) are given as trimethoprim MIC values.

Dilution ranges tested are framed by -. Isolates resistant to the highest tested concentration are given in the next concentration level (bold). Values for the lowest concentration include isolates with MICs below the tested range.

The majority (57%) of operations reported the application of antimicrobials as group medication during the fattening period. The reported spectrum of applied antimicrobials was broad, with tetracyclines (28%), macrolide/lincosamide antibiotics (25%) and beta-lactams (22%) constituting the most commonly administered classes of antibiotics in the survey. Up to five different substances were reported at farm level. MRSA was detected in 60% farms with reported, and in 42% with no reported antibiotic use at group level. Application of antibiotics was associated (OR: 2.1; CI: 1.3 - 3.4) with the presence of MRSA in the univariate analysis only (Table 1).

### Association of farm characteristics with test outcome

Eight factors (region, herd size, production type, flooring, purchase of pigs, use of antimicrobials, keeping of cattle in the farm, and animal flow system) with a significant univariate association with the test outcome based on a p-value < 0.05 were subjected to further Chi-squared tests to detect correlations among themselves. The correlation matrix revealed strong multicollinearity (data not shown), especially for the variable "region", which was then included as a fixed variable in the regression model. The multivariate logistic regression analysis included 273 cases with full information on the factors significantly associated with the test outcome (n = 8) according to Chi-squared test. The results of univariate and multivariate logistic regression are summarized in Table 1.

The multivariate model comprises the variables herd size, and production type. Large (> 1000 finishers) and medium-sized (500-999 finishers) farms were more likely to harbour MRSA compared to small operations housing less than 500 fatteners. Production types wean-to-finish and grow-to-finish were also associated with a higher likelihood of a MRSA finding in the herd. In the univariate analysis the purchase of pigs showed an impact on the MRSA test. A distribution of the positive MRSA results among farm characteristics according to the purchase of pigs is shown in Table 4. Operations that obtained their growers from specialized farms that purchase weaners to sell them as growers for fattening (weaner-to-grower producers), tested positive for MRSA (n = 5). Since only five operations in this purchase category took part in the study, and on account of the various origins of weaners in weaner-to-grower productions, the categories "purchase from > 2 sources" and "purchase from weaner-to-grower producers" were combined to the category "purchase from multiple sources" for logistic regression analysis.

**Table 4:** MRSA prevalence in herds with different properties according to the reported purchase of piglets

Variable	Category	No purchase No. (% pos.)	Purchase from 1-2 sources No. (% pos.)	Purchase from > 2 sources No. (% pos.)	Purchase from weaner-to-grower producers No. (% pos.)	Total No. (% pos.)
Herd size	Small	34 (21)	40 (38)	7 (29)	1 (100)	82 (31)
	Middle	18 (56)	48 (56)	16 (69)	4 (100)	86 (61)
	Large	19 (42)	75 (64)	21 (81)	0 (0.0)	115 (64)
	Total	71 (35)	163 (55)	44 (68)	5 (100)	283 (53)
Production type	Farrow-to-finish	53 (30)	8 (25)	3 (67)	0 (0.0)	64 (31)
	Wean-to-finish	9 (56)	25 (64)	3 (67)	1 (100)	38 (63)
	Grow-to-finish	10 (50)	132 (55)	37 (68)	4 (100)	183 (59)
	Total	72 (36)	165 (55)	43 (67)	5 (100)	285 (53)
Animal flow system	Continuous	36 (25)	43 (47)	9 (78)	0 (0.0)	88 (41)
	AIAO^a ^with cleanup	2 (0.0)	16 (44)	4 (75)	0 (0.0)	22 (46)
	AIAO^a ^with cleanup and disinfection	34 (50)	105 (60)	29 (62)	5 (100)	173 (60)
	Total	72 (36)	164 (55)	42 (67)	5 (100)	283 (53)
Use of antimicrobials^b^	No	45 (27)	64 (52)	15 (53)	0 (0.0)	124 (43)
	Yes	28 (50)	101 (57)	29 (76)	5 (100)	163 (61)
	Total	73 (36)	165 (55)	44 (68)	5 (100)	287 (53)

^a ^All in/all out

^b ^Reported treatments at group level

The final logistic regression analysis to test robustness showed that the variables herd size and production type had a stable predictive value in all seven data subsets (data not shown).

## Discussion

The present study reflects the wide dissemination of MRSA CC398 among German herds of fattening pigs. Additionally, farms that harbour more than 500 finishers, and wean- as well as grow-to-finish production types were identified as risk factors for a positive MRSA test result.

From a production chain point of view, the fattening sector takes a middle position between breeding pig herds and the slaughter pigs previously studied in Germany [19,20]. Although the three studies are not longitudinally connected, their results, and the pyramidal structure of pig production are in line with the vertical dissemination of MRSA from breeding (top) to production holdings (bottom; from fattening to slaughter pigs), when considered at herd level. Within herds, it has been observed that age has an impact on MRSA prevalence among pigs, and that there seems to be a strong decline in pig colonization towards the time of slaughter [23-25]. However, age-dependant differences in MRSA prevalence among finishing pigs were not addressed by our study design, since samples were taken within the same broad age group (10 - 30 weeks) among farms of different types.

In the present study, three farm production types were targeted. The prevalence for MRSA in wean- and grow-to-finish production holdings exceeded 58%. By contrast, farrow-to-finish operations tested only in 31% positive, on average. Multicollinearity among variables is a limitation of the present study that hampers the interpretation of the effect of a single factor on MRSA prevalence. The variable "production type" strongly correlated with the "purchase of pigs" (χ^2^, p < 0,001). The production types covered by this study differed in their purchase patterns of pigs for fattening. While wean- and grow-to-finish operations bought piglets in 76 and 95% of cases respectively, only 17% of farrow-to-finish herds purchased pigs. Furthermore, the size of herds is a risk factor for the presence of MRSA CC398 in a pig farm [26]. This reflects that intensive pig rearing implicates a greater pool of potential recipients. In the present survey, the prevalence of MRSA among middle-sized and large farms increased with the number of sources of pigs reaching 81% positives when pigs were purchased from multiple (> 2) sources. By contrast, the prevalence in small farms achieved a maximum of 38% when pigs were bought from one to two sources. Overall, middle-sized and large farms bought in 79 and 86% of cases pigs, compared to 59% of small farms. Chi-squared test also indicated a link between size of farms and purchase policy (χ^2^, p = 0,001). It has already been proposed, that MRSA is spread between farms via trade of colonized animals [27]. However, purchase alone does not explain a high MRSA prevalence, since grow-to-finish operations purchased pigs most frequently of all production types, but did not show the highest MRSA prevalence. It is most likely that certain trade flows are responsible for the circulation of MRSA CC398. Other drivers also may play a role in the spread of MRSA, such as an aerogen transmission in regions with high livestock density or vector borne transmission between farms. In addition, intra-herd factors should be investigated, since MRSA CC398 also occurs in closed herds implementing tighter biosecurity measures than in production holdings [20].

The collected data do not allow a sound comparison between regions, since the aimed sample size (n = 170) for each region was only reached in the southwest of Germany, in which the highest prevalence (59%) was observed.

Molecular characterization of the isolates mirrors the reported variety of strains in Europe [11,20,28]. The high diversity of resistance profiles detected is in line with previous investigations [16,17]. Some *mecA*-positive isolates were phenotypically susceptible to oxacillin. This has previously been reported and may in part be explained by heteroresistance [29]. To address this observation properly, additional investigations would be required which were not part of the present study. We identified a high proportion of resistant isolates to the combinations quinupristin/dalfopristin (32%) and sulfamethoxazole/trimethoprim (40%). Phenotypic resistance to quinupristin/dalfopristin (QUI/DAL) and sulfamethoxazole/trimethoprim in MRSA CC398 has already been reported, however, not at such high rates [16,30]. Resistance to the streptogramin A and B combination QUI/DAL is of concern since the compound represents an alternative to glycopeptides in therapy of human MRSA infections. Argudín et al. were not able to detect any of the genes coding for resistance to streptogramin A and B *vatA*, *vatB*, *vatC*, *vgaA*, *vgaB*, *vgaC*, *vgbA *and *vgbB *in 9 MRSA CC398 isolates phenotypically resistant to QUI/DAL [16], suggesting the isolates were not truly resistant or other mechanisms are responsible for resistance. The currently valid epidemiological cut-off for interpretation of resistance to QUI/DAL is quite low (≥2). Further analyses would be required to identify the underlying genetic mechanisms. Comparison of our results with MIC distributions for MRSA CC398 from previous studies reveals similar proportions of non-wild-type (resistant) phenotypes [1,30]. However, a high proportion of (83%) multidrug resistant isolates were found in this study, including resistance to the combinations quinupristin/dalfopristin and sulfamethoxazole/trimethoprim in the two most predominant MDR profiles. An association between certain resistance phenotypes and the widespread *spa*-type t011, as our results indicate, requires further clarification.

## Conclusions

The results of the present study reveal a high prevalence of MRSA CC398 in German fattening pig herds, mainly in large herds and wean-to-finish operations. The implication of pig trade and other factors in the spread of this microorganism requires further investigation since MRSA has been detected to a high extent in German breeding and closed breeding (nucleus) herds [20].

Identified SCC*mec- *and *spa-*types confirm circulating clones in the European pig industry. Susceptibility testing reveals a high amount of quinupristin/dalfopristin and sulfamethoxazole/trimethoprim non-wild-type phenotypes.

## Methods

### Survey design

In the period between May and December 2008, federal states of Germany were asked to collaborate in the present study to determine the spread of MRSA in herds of fattening pigs. Following the technical specifications in the course of the EU-wide survey in breeding pig herds as outlined in Commission Decision 2008/55/EC, the sampling plan aimed to collect five dust samples from different positions within the compartments with fattening pigs between 10 and 30 weeks of age of randomly chosen operations housing more than 100 finishers. Sample size was calculated based on an estimated prevalence of 50%, a 95% confidence interval, and 7.5% accuracy. To obtain representative results for Germany the sampling plan aimed to enrol a total of 510 herds located in the eastern (n = 170), north- (n = 170) and south-western (n = 170) regions of Germany, distributed within each region among federal states according to the proportion of fattening pig operations with > 100 animals. A questionnaire was used to determine the herd size, animal flow system, production type, flooring, health status in the herd, and application of antimicrobials as group medication in a four-month interval prior to sampling (fattening period), location of the operation, and housing of other animal species. Details of collected data are given in Table 1. The official veterinarian in charge of the region was assigned with the collection of the samples and completion of the questionnaire. Collected samples were shipped at room temperature within 10 days to accredited regional laboratories or optionally to the National Reference Laboratory for Coagulase Positive Staphylococci incl. *S. aureus *(NRL Staph) at the BfR for primary isolation of MRSA 13 days after sampling at the latest. Sample collection, shipping and storage complied with the methods described in Commission Decision 2008/55/EC.

Primary isolates obtained at regional laboratories were sent for confirmation, further typing and antimicrobial susceptibility testing of MRSA to the NRL Staph.

### Bacterial culturing

Primary isolation was performed in all laboratories as follows and in accordance with Commission Decision 2008/55/EC. Samples were pooled per farm and cultured for pre-enrichment in Mueller Hinton broth supplemented with 6.5% NaCl. After incubation for 16-20 h at 37°C, 1 ml was inoculated into 9 ml of tryptic soy broth containing 3.5 μg/ml cefoxitin and 75 μg/ml aztreonam, and incubated again at 37°C for 16-20 h. A loopful was then plated onto sheep blood agar and chromogenic MRSA screening agar (Brilliance MRSA Agar, Oxoid, Wesel, Germany, and/or CHROMagar MRSA, Mast Diagnostica, Reinfeld, Germany). After incubation for 24-48 h at 37°C regional laboratories sent five presumptive colonies as subcultures to the NRL Staph for confirmation.

### SCC*mec*- and *spa*-typing

DNA extraction was performed using the "RTP^® ^Bacteria DNA Mini Kit" (Invitek, Berlin, Germany). MRSA isolates were confirmed by multiplex PCR targeting 16S rRNA-, *nuc*-, and *mecA*-genes, as described previously [31].

Further characterization of the isolates involved SCC*mec*-typing for the identification of types I-V [22], and *spa*-typing [32]. One MRSA isolate of each positive farm was SCC*mec*- and *spa*-typed. A multilocus sequence type (ST) was determined for selected *spa-*types [33]. Sequencing of PCR products was conducted by Agowa, Berlin, Germany.

*Spa*-types were assigned using the Ridom StaphType software (Ridom GmbH, Würzburg, Germany), and MLST sequence analysis was carried out by matching with *S. aureus *MLST database (http://saureus.mlst.net).

### Antimicrobial susceptibility testing

Susceptibility tests were carried out using the broth microdilution method according to CLSI guidelines [34]. Custom-made microtitre plate panels were used (TREK Diagnostic Systems, Magellan Biosciences, West Sussex, England). Overall, susceptibilities to 13 antimicrobial agents/combinations of agents were determined and for each, 5-8 concentrations in 2-fold dilution series were tested (Table 3). *S. aureus *strain ATCC 25923 was used as a control. Evaluation of resistance was based on epidemiological cut-off values published by the European committee for antimicrobial susceptibility testing for MRSA and *S. aureus *(http://www.eucast.org).

### Statistics

Data analysis was performed using the statistical software program SPSS 18.0 (SPSS Inc. Munich, Germany). Univariate analysis was performed using Chi-squared test to select for herd parameters significantly associated (p-value < 0.05) with the test outcome, and to estimate correlations among variables. Multivariate analysis of the data included backward stepwise binary logistic regression with the test result as the outcome (negative = 0/positive = 1) and the herd characteristics as categorical covariates to estimate odds ratios (OR) including those variables significantly associated with the outcome in the univariate analysis. For this purpose, only cases with complete information on variables included in the model were considered.

To test the robustness of the model, the original sample (n = 290) was randomly divided into seven sub-samples (~41 cases each), and then subjected seven times to stepwise binary logistic regression each time excluding one of the subsamples.

## Authors' contributions

KA carried out data analyses and drafted the manuscript. AF participated in the microbiological testing of the strains. JAH and SH carried out the molecular genetic studies. AS and BG were responsible for susceptibility testing of the strains. NS, AG and CMG investigated statistical analyses made by KA. JB, AK, AH and BA contributed to the drafting of the manuscript. BAT is responsible for study design and coordination, and helped to draft the manuscript. All authors read and approved the final manuscript.
